# Validation of Diagnostic Methods for European Foulbrood on Commercial Honey Bee Colonies in the United States

**DOI:** 10.1093/jisesa/ieab075

**Published:** 2021-11-01

**Authors:** Meghan O’Grady Milbrath, Peter Daniel Fowler, Samuel K Abban, Dawn Lopez, Jay D Evans

**Affiliations:** 1 Department of Entomology, Michigan State University, 4090 N. College Road, Pollinator Performance Center, RM 100, Lansing, MI 48910, USA; 2 Department of Comparative Medicine and Integrative Biology, Veterinary Medical Center, Michigan State University, 784 Wilson Road, Room G100, East Lansing, MI 48824, USA; 3 Bee Research Laboratory, USDA–Agricultural Research Service, B306 BARC-East Beltsville, MD 20705, USA

**Keywords:** European foulbrood, lateral flow device, honey bee, diagnostics

## Abstract

One of the most serious bacterial pathogens of Western honey bees (*Apis mellifera* Linnaeus [Hymenoptera: Apidae]) is *Melissococcus plutonius*, the cause of the disease European foulbrood. Because European foulbrood is highly variable, with diverse outcomes at both the individual and colony levels, it is difficult to diagnose through visual inspection alone. Common lab diagnostic techniques include microscopic examination and molecular detection through PCR. In 2009, a lateral flow device was developed and validated for field diagnosis of European foulbrood. At the time, *M. plutonius* was thought to be genetically homogenous, but we have subsequently learned that this bacterium exists as multiple strains, including some strains that are classified as ‘atypical’ for which the lateral flow device is potentially less effective. These devices are increasingly used in the United States, though they have never been validated using strains from North America. It is essential to validate this device in multiple locations as different strains of *M. plutonius* circulate in different geographical regions. In this study, we validate the field use of the lateral flow device compared to microscopic examination and qPCR on larval samples from 78 commercial honey bee colonies in the United States with visual signs of infection. In this study, microscopic diagnosis was more sensitive than the lateral flow device (sensitivity = 97.40% and 89.47%, respectively), and we found no false positive results with the lateral flow device. We find high concurrence between the three diagnostic techniques, and all three methods are highly sensitive for diagnosing European foulbrood.

Honey bees (*Apis mellifera* Linnaeus [Hymenoptera: Apidae]) are susceptible to a variety of viral and bacterial diseases. One of the most severe honey bee diseases is European foulbrood (EFB), caused by the bacterium *Melissococcus plutonius* (White 1912, Bailey 1957). Infection with this pathogen can lead to losses of colonies and honey crops as well as increased costs in labor, equipment, and treatments. Direct economic impacts of EFB have been recently estimated to range from $300 to $500 per hive (Laate et al. 2020), and indirect economic impacts are much larger, as honey bees provide the bulk of pollination services to U.S. crops, valued at $34 billion (Jordan et al. 2021). Infection rates of EFB appear to be rising globally (Wilkens and Brown 2007, Roetschi et al. 2008), though the reasons for this resurgence are not understood, European foulbrood remains a disease of national and global concern for honey bees (Ellis and Munn 2005, Boncristiani et al. 2021).

Only one treatment is labeled for the management and control of EFB in the United States, the broad-spectrum antibiotic oxytetracycline. This antibiotic previously was available directly for purchase by beekeepers, but recent federal regulation now requires that beekeepers obtain an order from a licensed veterinarian to purchase any honey bee antibiotics (FDA, 2015). While this regulation is an important step toward better antibiotic stewardship across the food animal industry, it has made the treatment of EFB and other diseases more difficult, as most veterinary professionals lack training and experience in diagnosing diseases of honey bees. Historically, diagnosis of EFB has relied on visual identification of pathologic signs (Forsgren 2010; and Forsgren 2013). Visual identification, however, can be difficult for inexperienced beekeepers and veterinarians, especially if infection is mild, as many of the signs are non-specific for EFB. Both beekeepers and veterinarians are concerned about misdiagnosis of EFB and other honey bee diseases.

To further complicate field diagnosis, EFB disease is highly variable in terms of both colony outcomes and visual signs. Outcomes vary widely within affected apiaries with some colonies succumbing to disease while others appear completely asymptomatic (Forsgren et al. 2005, Belloy et al. 2007, Roetschi et al. 2008, Budge et al. 2010). Outside factors are thought to play an important role in colony outcome after EFB infection, including concurrent stressors related to food, weather, and brood nest growth (Bailey 1961, Forsgren 2010); overall bacterial loads (Budge et al. 2010); specific co-infections (Erban et al. 2017, Lewkowski and Erler 2019); pesticide exposure (Wood et al. 2020); or differences in microbiota (Erban et al. 2017, Floyd et al. 2020). Some of this variability in virulence and colony outcome may be due to the presence of secondary bacterial pathogens, including *Paenibacillus alvei, Enterococcus faecalis, Brevibacillus laterosporus,* or *Achromobacter eurydice* (Bailey 1957, Forsgren 2010, Erler et al 2018). It is not known if these bacteria increase virulence or lethality of *M. plutonius*, or if they colonize dead and dying larvae (Charriere et al. 2011, Lewkowski and Erler 2019, but either way, their presence may alter the visual presentation of infected larvae and make field diagnoses more difficult. Visual identification can be further hampered by the presence of other diseases, including chalkbrood and the disease complex associated with the parasitic mite *Varroa destructor.*

*Melissococcus plutonius* itself is known to be highly variable in terms of virulence, with some strains killing a higher proportion of hosts and killing their hosts in a shorter time period (Arai et al. 2012, Grossar et al. 2020). Young larvae that die early from infection retain their shape on the bottom of the cell, and their trachea are often visible as their bodies flatten and become transparent. Infected larvae can also survive until the pre-pupal stage, dying after capping, resulting in a sunken capped cell, reminiscent of the signs of another bacterial disease, American foulbrood. Infected older larvae change dramatically as they die. Early infection causes slight color changes and malpositioning of the larvae, often in a twisted or corkscrew manner. In later infection, the larvae generally lengthen along the lower cell wall and their bodies lose pressure, giving them the visual appearance of melting and loss of segmentation. As decay progresses, the larvae can become further discolored, ranging from brown to greyish black, ultimately drying to a dark, rubbery scale (Forsgren 2013). All of these stages can be found simultaneously in the hive during a single outbreak.

Field diagnosis of EFB can be confirmed through a number of laboratory methods. Microscopic visualization of lanceolate cocci forming pairs or chains can be easily performed with either carbol fuchsin or gram stain preparation (Forsgren 2013). Additional laboratory tests have been developed with high sensitivity and specificity for the detection of *M. plutonius* including ELISA (Pinnock and Featherstone 1984), PCR (Govan et al. 1998), hemi-nested PCR (Djordjevic et al. 1998, McKee et al. 2003), and qPCR (Evans 2006, Roetschi et al. 2008). Laboratory diagnostic tools are an important component of diagnoses but they are often time consuming and expensive, making them impractical for informing treatment options in the field. In 2009, Tomkies et al. developed and validated a novel field test kit for diagnosing EFB, which is now widely available and marketed by VITA (Europe) Ltd. This test uses a lateral flow device (LFD) containing monoclonal antibodies derived from mice immunized with *M. plutonius.* Tests are performed on a single larva and can be done in the field in less than 10 minutes. Laboratory validation of the initial prototype was performed in both the United Kingdom and Italy with promising results (96.4–100% sensitivity and 100% specificity). Field trials were also conducted and showed similar results to laboratory diagnostics (96.8% sensitivity and 98.9% specificity; Tomkies et al. 2009).

At the time the lateral flow device was developed, *M. plutonius* was thought to be a homogenous group of bacteria with little variation between strains or isolates (Bailey and Gibbs 1962, Allen and Ball 1993, Djordjevic 1999, Forsgren 2010). However, recent molecular characterizations have painted a more complicated picture, identifying 37 strains belonging to three different clonal complexes (Okumura et al. 2011, Arai et al. 2012, Haynes et al. 2013, Budge et al. 2014, Takamatsu et al. 2014, Djuikic et al. 2018, de León-Door et al. 2018, Okumura et al. 2018, Okumura et al. 2019). The distribution of these strains varies geographically, with different strains dominating infections in different countries and regions (Budge et al. 2014, Takamatsu et al. 2014, Erban et al. 2017, de León-Door 2020).

Following reports in Japan of *M. plutonius-*like bacteria with different phenotypic characteristics, biochemical and molecular analysis revealed a unique group of *M. plutonius* strains categorized as ‘atypical’ (Arai et al. 2012). Unlike typical strains, atypical strains were shown to be non-fastidious, able to grow in aerobic conditions and on media without potassium salt supplementation. These strains were initially suspected to be restricted to Japan (Takamatsu et al. 2012), but have since proven to be widespread (de León-Door et al. 2018): to date, atypical strains have additionally been reported in England and Wales, Brazil, Netherlands, United States (Haynes et al. 2013), Mexico (de León-Door et al. 2018), and Canada (Woods et al. 2020). Laboratory testing with the lateral flow device in Japan showed that the device worked as expected with typical strains but responded only weakly to extracts from larvae infected with atypical *M. plutonius* (Takamatsu et al. 2012). It is therefore not known if the lateral flow device will work effectively for strains of *M. plutonius* circulating in the United States, and to date, we are not aware of any publications indicating that this test has been validated on the North American continent.

Here we present a validation study of diagnostic methods of European Foulbrood with samples from honey bee hives from multiple commercial operations in the United States that showed EFB symptoms in the field. We validate the lateral flow device test and compare the results with both microscopy and molecular quantification of *M. plutonius,* and we evaluate sensitivity of the test in the presence of secondary bacteria. Validation of diagnostic methods, including a field relevant test is essential for the diagnosis and treatment of European Foulbrood disease and the protection of honey bee health.

## Materials and Methods

### Sample Collection

We tested a total of 390 larval samples from 77 honey bee colonies originating from 13 different apiaries in Michigan, USA. All honey bee larval samples were collected May–June 2019 from commercial honey bee colonies in that were monitored as part of a larger cohort study. These honey bee colonies were from three different commercial beekeeping operations and had recently been brought into Michigan from southern states where they had been managed after California almond pollination. Larval samples were taken from each colony where any visual signs of European foulbrood were observed, including yellowish color, visible tracheae, and malpositioning of older larvae. Disease severity ranged from mild (less than five larvae with visible signs of disease) to severe (over 100 larvae with visible signs of disease), and all colonies were in yards with multiple other colonies exhibiting visual signs of EFB. In each diseased hive, we sampled five older larvae by using a cleaned forceps, placing the larva in a labeled individual microcentrifuge tube. If fewer than five diseased larvae were observed, we took all that were present. We selected older larvae (fourth or fifth instar) that were just starting to show signs of disease (malpositioning in the cell or discoloration). We did not select larvae that were severely decayed. Samples were placed on dry ice in the field and stored at −20°C until shipment to the USDA-ARS Bee Research Laboratory for testing. Samples from each colony were tested in three ways: microscopic evaluation for the presence of *M. plutonius* and *Paenibacillus. alvei*, presence of *M. plutonius* using the Vita Lateral Flow Device (LFD), and qPCR for *M. plutonius*.

### Microscopic Analyses

Larval samples were pooled together by hive in a microcentrifuge tube (between 1 and 5 symptomatic larvae per hive). An aliquot of the larval suspension was prepared for microscopic analysis using the modified hanging drop method (Shimanuki and Knox 1991) and stained with Carbol fuchsin stain for 10 s. The sample was examined using an oil immersion objective at 100× magnification. Diagnosis was made based on the morphological characteristics of *M. plutonius* (the presence of lanceolate cocci cells that occur singly, in short chains, or in pairs and measures 0.5–0.7 × 1.0 µm). The larval suspension was also analyzed for *P. alvei* which is commonly detected as a secondary spore forming bacteria in larvae infected with *M. plutonius*.

### Lateral Flow Test

A small aliquot (50 µl) of the pooled larval suspension prepared for microscopy was transferred into the extraction buffer solution provided in each LFD kit and shaken for 20 s, per kit instructions. Three drops of the mixture were transferred into the LFD test well. The indicator lines on the LFD test kit were read after 3 min. Double lines (Test and Control) indicate a positive confirmation of *M. plutonius*, whereas a single line (Control) indicates the absence of *M. plutonius* in the sample.

### Quantitative PCR Analyses

We performed qPCR analyses on each individually sampled larvae (*N* = 390) as well as on the pooled samples (by hive). We tested each individual and pooled sample for *M. plutonius* by qPCR using primers for a 271-base-pair section of the 16S rRNA gene (Evans 2006) and standard procedures for qPCR quantification using SsoAdvanced Universal SYBR Green Supermix (Evans et al. 2013). Because we were screening for disease, we chose the more conservative Cq threshold of 40. Although the input material (five larvae) was identical and consistent between tests, we did not use a honey bee reference gene to normalize DNA amounts, and therefore, the results are semi-quantitative. Because there is no reason to anticipate bias or trends in our samples (e.g., by apiary), and because of our high numbers of samples, the resultant noise would not affect trends in the results.

All statistical analyses were carried out using R version 4.0.4 (R core team 2021). Bacterial loads between different groups were compared using an unpaired student t-test. Odds-ratios were used to determine possible impacts of *P. alvei* in pooled samples on the results of the LFD.

## Results

### Sensitivity

Almost all of the samples tested positive for *M. plutonius* by qPCR: of the 390 individual samples, 374 were positive for EFB by qPCR and 16 were negative using 40 qPCR cycles as the cutoff. Every hive had at least one larval sample that had detectable levels of *M. plutonius* present. Nine hives had at least one larva that was negative (ranging from 1 to 4 negative larvae per hive). Of the 78 pooled samples (all larvae from each hive pooled together), two were negative by qPCR, and one sample was excluded for analysis by error. Individual larval samples from the two qPCR-negative colonies did reveal the presence of *M. plutonius*, albeit at levels that were only slightly above the cutoff for a negative diagnostic: one colony had two negative larval samples and three larval samples with very low levels (Cq = 3.39, 1.99, 1.6). The other colony had four individual larvae testing negative by qPCR, and one sample with very low levels (Cq = 1.72). Notes by the sample collectors at the time of collection indicate that the former negative hive had only a few malpositioned larvae, and that it could not be visually determined if they were related to EFB or early chalkbrood infection. Two weeks prior to collection, this colony had appeared healthy, and it was not inspected after larval collection. The second hive had severe chalkbrood infection at the time of sampling (over 10 mummies visible in cells), but 2 wk prior to sampling also had severe signs of EFB (multiple melted larvae and larvae with visible trachea).

The two pooled samples that were qPCR negative were also both negative by the lateral flow device, but one was positive by microscopy (the hive that had had prior severe EFB and current chalkbrood). The lateral flow device also registered eight additional negative results which were positive by qPCR. Three of these eight LFD-negative samples were also found negative by microscopy (Table 1). There were no samples that were positive by the lateral flow device but were negative by either microscopy or qPCR. In this study, microscopic diagnosis was more sensitive than the lateral flow device (sensitivity = 97.40%, 95% CI: 90.93–99.68 for microscopy; sensitivity = 89.47%, 95% CI: 80.31–95.34% for LFD), but both tests were highly sensitive for EFB.

**Table 1.** Results by diagnostic method using five larval samples from each hive

	Microscopy	Lateral flow device	qPCR
Negative	3	10	2
Positive	75	68	75

### Bacterial Load

**Fig. 1.**
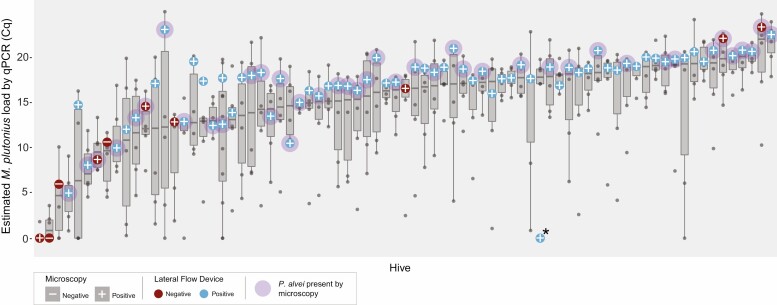
Estimated load of *M. plutonius* by qPCR. Each box plot represents an individual hive, and each point represents an individual larval sample. The large circle indicates the level of the pooled sample, except for one point (indicated by an asterisk *) in which the qPCR was not performed. The line in the box represents the median of the five samples. The plus/minus sign in the center of the circle indicates the result by microscopy (plus = positive, minus = negative), and the color of the circle indicates the result by the lateral flow device (blue = positive, red = negative). A purple halo is around the circle for samples where *P. alvei* was found.

Bacterial load varied both within hives (by larva) and among hives (Fig. 1). Quantification cycle (Cq) in all individual samples ranged from 0 to 23.55 and on average were 13.14. One colony that contained the larval sample with the highest bacterial load (Cq = 23.55) detected in this project also had a larva with no detectable *M. plutonius*. Of the 374 positive individual larval samples, 34 had very low levels (Cq ranging from 0.27 to 4.98). Pooled samples Cq values ranged from 0 to 21.93 with an average of 15.36. The pooled samples that tested negative by microscopy Cq ranged from −0.73 to 9.98. The mean Cq for the three samples negative by microscopy (4.95) was lower than the mean Cq of the samples that were positive for microscopy (15.77) (*t* = 4.34, df = 75, *P* < 0.0001). The mean of the pooled samples (15.36) was higher than the mean of all of the individual samples taken together (13.14, *t* = 3.195, df = 465, *P* = 0.0015). One sample had high bacterial levels in all larval samples, but qPCR was not performed on the pooled sample. The data point was included in the figure to show the positive results by microscopy and LFD but not included in statistical analysis.

The samples that were negative by microscopy had low bacterial loads, whereas the samples that were negative by the LFD had a broad range of bacterial loads (Fig. 1). The three samples that were positive by qPCR but negative by microscopy had Cq values of −0.73, 5.61, and 9.98. The LFD-negative samples included the samples negative by microscopy, as well as seven other samples ranging up to Cq 21.93 (mean 10.71).

### Presence of Secondary Bacteria

**Fig. 2.**
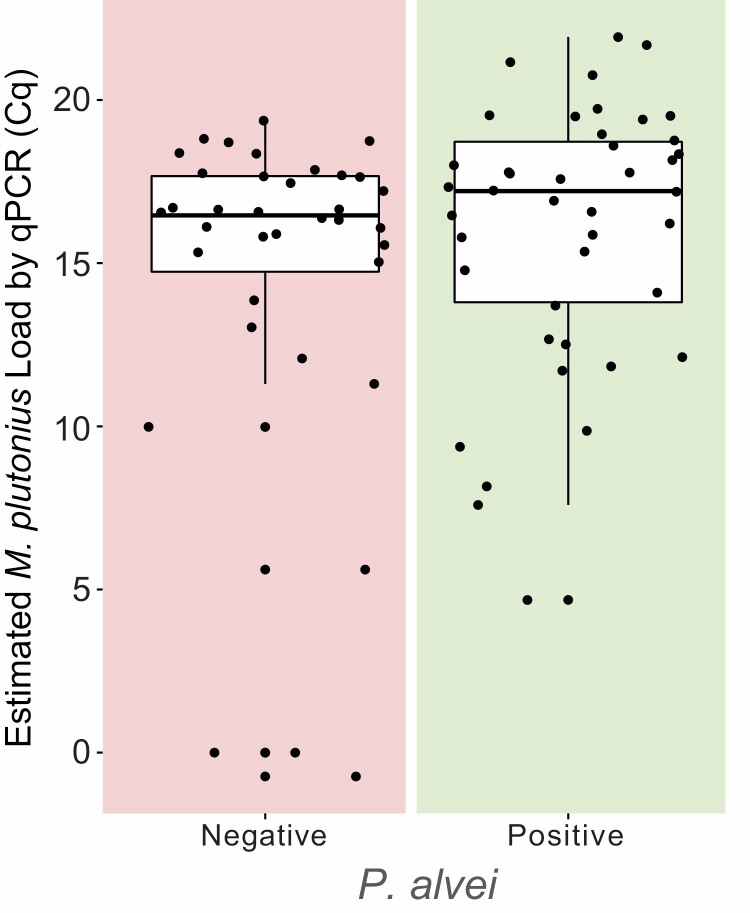
Estimated bacterial load of *M. plutonius* for pooled larval samples based on the presence (right panel) or absence (left panel) of *P. alvei.*.

*Paenibacillus alvei* was found in 42 of the 77 pooled samples. All of the samples with *P. alvei* were positive for *M. plutonius* by qPCR and by microscopy, but four of these samples were negative by the LFD. As shown in Fig. 2, there is no difference in the mean Cq of *M. plutonius* in samples that had *P. alvei* (16.02) compared to samples where *P. alvei* was not identified (14.56) (*t* = 1.36, df = 75, *P* = 0.1778). Within the samples that were negative by the LFD, the mean Cq for those containing *P. alvei* (16.1375) is higher than those with no *P. alvei* detected (7.0833), but due to low sample size (*N* = 10), the difference is not significant (*t* = 2.14, df = 8, *P* = 0.0646). The presence of *P. alvei* is not associated with a higher likelihood of a positive result with the LFD (OR = 1.27, 95% CI = 0.29–5.49).

## Discussion

All three diagnostic methods, microscopy, qPCR, and the lateral flow device, were sensitive to the identification of *M. plutonius* in larvae from colonies exhibiting visual signs of European foulbrood in Michigan. While the sensitivity of the LFD was relatively high, it is interesting to note the number of false negatives (8 of 78). Some of these false negatives also showed high loads (Cq = 21.93) of *M. plutonius* by qPCR. It is therefore important for beekeepers and veterinarians to understand the limitations of the LFD device in the field and to recognize that a negative LFD result does not necessarily indicate the absence of *M. plutonius* or European foulbrood disease.

This study also importantly demonstrates that pooled larval samples can be used for both qPCR detection and diagnosis with the LFD. Overall, there was good concordance between the 5 individual samples per hive and the pooled sample for all tests. One pooled sample that was negative by qPCR did have an individual larva that tested positive by qPCR. At the time of sampling, this hive contained only a few larvae that were malpositioned, but did not, at the time, have an obvious case of EFB by visual diagnosis, nor did it develop other signs of EFB that were noted during other inspections carried out as part of the larger cohort trial. The presence of larvae with a positive result for *M. plutonius* in the absence of colony-wide disease supports other work that indicates that qPCR, while important for screening for the presence of pathogens, may be too sensitive to indicate which colonies will necessarily develop European foulbrood disease (Sopko et al. 2020). Further work is required to identify the specificity of these tests and to relate molecular results to eventual disease. Our results, however, indicate broad similarity in diagnostics when symptomatic larvae are sampled, a routine commonly requested for diagnostics services (e.g., https://www.ars.usda.gov/northeast-area/beltsville-md-barc/beltsville-agricultural-research-center/bee-research-laboratory/docs/how-to-submit-samples/).

Interestingly, the one case in which the pooled sample was negative by qPCR but positive by microscopy also had severe chalkbrood infection (caused by the fungus *Ascosphaera apis)*. While it is easy to identify chalkbrood in larvae once they are in the mummy stage, it is difficult to identify larvae that are in the dying process; in both EFB and chalkbrood, larvae can take on a general melted appearance. It may have been the case that the diseased larvae that were sampled for this study were malformed because they had chalkbrood, and not by EFB, though this was not confirmed for this sample. This colony had severe EFB infection (visual diagnosis) during a previous inspection.

Here we used the more conservative Cq cutoff of 40 to determine the presence of *M. plutonius*. Using a less conservative cutoff of 35 did little to increase the specificity of our results, as only one pooled sample (5 larvae) was between 35 and 40. Exclusion of this sample would change the reported sensitivity of the LFD from 89.47% (95% CI :80.31–95.34%) to 89.33% (80.06–95.28%). Sensitivity for microscopy would change from 97.40% (95% CI: 90.93–99.68) to 97.37% (95% CI: 90.82–99.68). This pooled sample, however, had multiple individual larval samples that were lower than 35Cq, and the colony had positive visible signs of disease supporting our use of the more conservative screening cutoff of 40Cq.

During field sampling, we chose larvae that were only lightly symptomatic, though these samples may have come from colonies that had larvae in various stages of disease and decay. We did not select samples that were severely decayed, nor did we use any scales. Because it is likely that beekeepers and veterinarians will use a variety of larval samples, and may even select for larvae that look more severely diseased (e.g., darker color or less body definition), more work should be done to validate diagnostic tests over a wider range of larval condition. Importantly, we demonstrate that all three diagnostic techniques are robust to the presence of the associated bacteria *P. alvei.* It is not certain if this pathogen is present at the time of infection, or invades later, and much more research is required to understand the bacterial dynamics occurring during larval decay.

Importantly, we took samples only from colonies that presented signs of larval disease, and we did not test colonies that were apparently healthy. It has been shown that bees in clinically healthy colonies can carry *M. plutonius* (Belloy et al. 2007, Budge et al. 2010, Erban et al. 2017). Further analysis of these testing methods would be useful to determine the specificity of each test as well as their utility as screening tools. Further work should also be done to evaluate different techniques in molecular detection. We chose to use 16S RNA based on its widespread previous use (Budge et al. 2010, Rivière et al. 2013, Floyd et al. 2020, Sopko et al. 2020), and because16S rRNA primers have been screened against other pathogens known to be present in brood that may produce false positives, and it has been found to be highly specific for *M. plutonius* (Govan et al. 1998, Sopko et al. 2020). It would be worth investigating other single-copy genes such as the sodA gene (Nesvorna et al. 2020, Sopko et al. 2020).

Given previous observations that atypical strains may show only weak positives by LFD (Takamatsu et al. 2012), our negative LFD results may possibly be due to strain variation. The LFD was developed using a monoclonal antibody against a single strain of *M. plutonius*, and these antibodies may not be as sensitive for detecting genetically distinct strains (Tomkies et al. 2009). Further strain characterization may help elucidate the effect that strain variation has on diagnostic results. The primers used for *M. plutonius* have been vetted against other bacteria species (Evans 2006) but more work needs to be done to test isolates from typical and atypical strains of *M. plutonius* and from related symbionts found in honey bee larvae. Future work will involve further improvement of primer design in light of recent attempts to more fully understand the genomic variation of the causative agent (Floyd et al. 2020).
